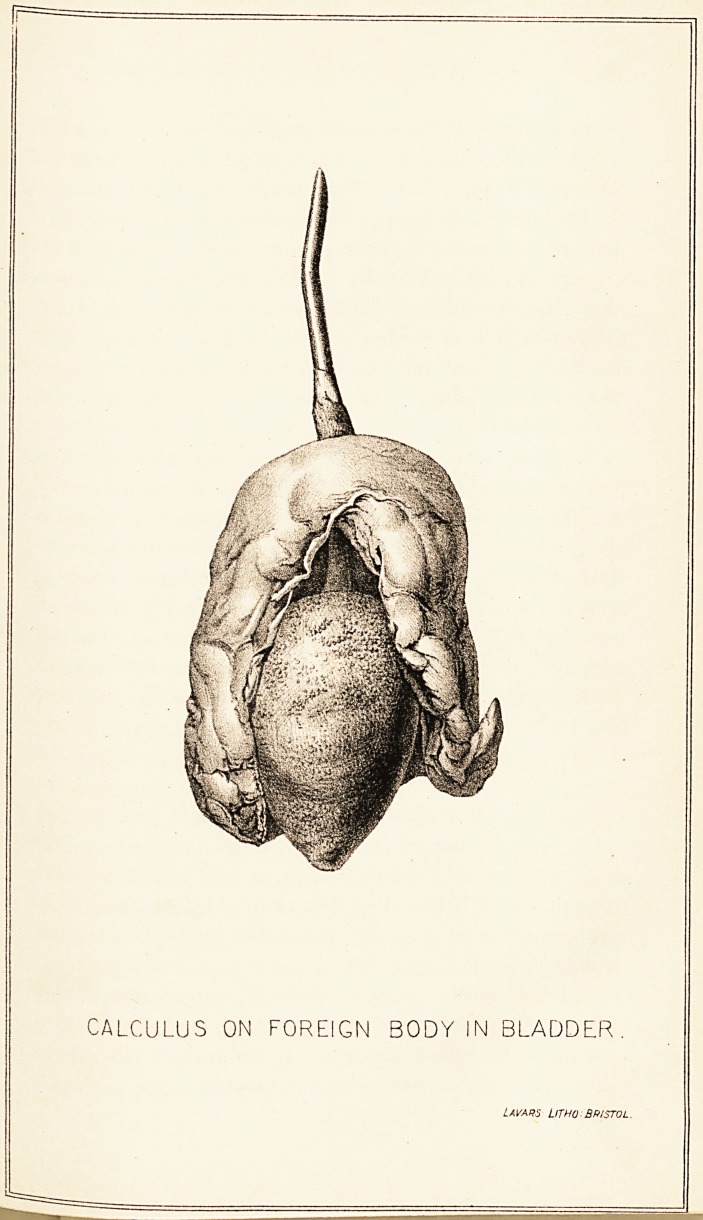# Calculus on Foreign Body in Bladder; Perforation of Bladder. No Symptoms

**Published:** 1886-03

**Authors:** H. Benham, J. Greig Smith

**Affiliations:** Assistant Medical Officer, Bristol Lunatic Asylum


### CALCULUS ON FOREIGN BODY IN BLADDER; PERFORATION OF BLADDER. NO SYMPTOMS. By H. Benham, M.D. (Assistant Medical Officer, Bristol Lunatic Asylum), and J. Greig Smith.

Curpty, T. H., seaman, æt. 21, single, was admitted into the Bristol Lunatic Asylum on the 14th April, 1881, suffering from "dementia." Nothing occurred to call for special note until 7th February, 1883, when he had an epileptic fit, the first he had ever had; and from this date he continued to have them at short intervals. On October 10th, 1883, I was called to see him about 10 a.m., and found him in bed, with a stream of blood issuing from the mouth of the urethra.

The Medical Superintendent, Dr. Thompson, and I carefully examined him, when a bruise, 2 inches in length and 1 in breadth, was discovered on the right side of the perineum. Catheterism was tried, but failed; and he was seen at 2 p.m. by Mr. Greig Smith. Attempts to pass a catheter proving unsuccessful, and the bladder having become distended, Mr. Greig Smith performed perineal section. On making the necessary incision, the urethra was found to be completely severed, lying in a mass of blood-clot, and the neighbouring parts very much bruised. The central end of the urethra was found collapsed into a little tag of fibrous tissue; a gum elastic catheter was introduced into the bladder; its ivory head was cut off, and it was carried from behind forwards through the penile urethra, and retained in position by strapping.

Patient made a statement that while working in the garden, at 3 p.m. on the previous day, when stooping to pick up potatoes, an attendant came behind and gave him a violent kick. He progressed satisfactorily, and his temperature did not reach 100° until two weeks after the injury, when the temperature rose to 101° and his abdomen became tense, and he complained of pain over the ileo-cæcal region. The next day, Mr. Greig Smith removed the catheter, which was coated with phosphates; and in a day or two patient was about as usual, and resumed work in the garden.

On 27th February, 1884, four months afterwards, symptoms of retention appeared. Mr. Greig Smith again saw him, and with some difficulty passed a small silver catheter, which was tied in. Next day patient succeeded in removing the catheter, and complained of pain over the fundus of the bladder; but shortly became as usual, and recommenced garden work. He had an attack of epileptic excitement in June, 1885; but with that exception kept well, and continued to work in the garden until 10th of October last, when he relapsed into the "status epilepticus" and gradually sank, and died four days later, on 14th October, 1885. As it is our practice to make *post-mortem* examinations where the friends do not object, I proceeded to do so, and the death was certified as being due to epilepsy and chronic Bright's disease, the signs of the latter being well marked.

The following extract from the *post-mortem* notes will explain the condition noticed in the specimen:

"On pulling the intestines aside, a grooved piece of iron was seen to be projecting from the vertex of the bladder into the abdominal cavity for a distance of two inches. This was slightly bent at its terminal inch, and lay between the coils of the small intestine, which was slightly congested, and at one or two points united by slight adhesions. The entire length of the foreign body, which appears to be part of the rib of a paragon-framed umbrella, is $4\frac{1}{8}$ inches. That portion of it remaining in the bladder was found to be coated over with a phosphatic deposit, forming a pear-shaped calculus, $1\frac{7}{8}$ inch in its vertical and $1\frac{1}{4}$ inch in its greatest transverse diameter. *(See drawing.)* The bladder was contracted and its coats thickened. On dissecting out the urethra, a stricture was found in the membranous portion, 1 inch in length, through which it was just possible to pass the blunt end of a *post-mortem* needle."

Death was evidently not due to or accelerated by the presence of the foreign body. The patient was a lost and demented epileptic, and belonged to a class of cases which are notorious for their tolerance of pain and ability to bear with but slight apparent inconvenience what to others would cause the most acute suffering. He, in all probability, picked up and secreted the piece of umbrella frame while working in the garden, and some time or other pushed it up his urethra and forced it into the bladder, and thence into the abdominal cavity.

A remarkable feature in this extraordinary case is the little peritonitis set up in the abdominal cavity, and the small amount of inconvenience experienced by the patient —the man continuing to work as usual until within four days of his death, which took place just over two years from the time of the alleged injury. The attendant who was suspected of kicking the patient was tried for the offence, but was acquitted from lack of evidence.

*Remarks by Mr. Greig Smith.*—My explanation of this case is, that the patient, beginning to find difficulty in passing water as the stricture contracted, sought to relieve himself by passing an instrument as he had seen

CALCULUS ON FOREIGN BODY IN BLADDER.

LAVARS LITHO: BRISTOL.

us do for him. Such an instrument could scarcely have produced the original injury, with complete division of the membranous urethra and large extravasation of blood. A vessel of considerable size was found spouting deep in the wound, and the point of the instrument, which is rounded and blunt, could not have divided this vessel.

I have on four occasions had to operate for complete rupture of the urethra in the perineum. The central end of the urethra I have always found as a piece of shrivelled fibrous tissue, lying in the midst of the extravasated clot. When the clot has been removed, the finger placed under the symphysis pubis will make out the normal urethra; following this forwards, the torn end is detected, caught in tenaculum forceps, and the crushed tissues unravelled and the opening found. A soft catheter is passed into the bladder; the head of it is cut off and passed through the urethra from behind forwards, and the instrument left in the bladder. If there is no urinary extravasation, deep and superficial suturing may be advisable; if there is, the wound is best left open. The catheter passed from the perineum to the meatus, is made to follow another instrument as guide passed in the usual way.

This is an example of Poulet's* fourth type of foreign body in the bladder—the most common of all. The instruments are long and sharp—pins with ivory or glass heads, hairpins, bodkins, and such-like. The points of the foreign bodies are rarely involved in the calculous incrustation. This is partly due to the fact that they often penetrate the bladder. In this case the foreign body passed through the bladder into the peritoneal cavity for a distance of two inches, apparently without doing any

* *A Treatise on Foreign Bodies in Surgical Practice*, vol. ii., p. 153.

harm. Phosphatic deposit was laid down on the metal in slight amount for half-an-inch outside the bladder, and this suggests that the walls of the bladder, as it filled and emptied, slipped up and down the instrument, giving opportunity of only intermitting incrustation. It is nothing short of marvellous that no escape of urine into the peritoneum should have taken place.

Supposing the stone had been detected, its removal would have been by no means free from danger. With the stone the foreign body must have come away, and a bladder fistula left opening into the peritoneum. Abdominal section and suturing of the hole in the bladder would then have been indicated.

Poulet speaks of death as the universal result of perforation of the bladder by a foreign body. Here is a case in which life continued over many months, and was not apparently shortened by the perforation.

## Figures and Tables

**Figure f1:**